# Including trait-based early warning signals helps predict population collapse

**DOI:** 10.1038/ncomms10984

**Published:** 2016-03-24

**Authors:** Christopher F. Clements, Arpat Ozgul

**Affiliations:** 1 Department of Evolutionary Biology and Environmental Studies, University of Zurich, Zurich CH-8057, Switzerland

## Abstract

Foreseeing population collapse is an on-going target in ecology, and this has led to the development of early warning signals based on expected changes in leading indicators before a bifurcation. Such signals have been sought for in abundance time-series data on a population of interest, with varying degrees of success. Here we move beyond these established methods by including parallel time-series data of abundance and fitness-related trait dynamics. Using data from a microcosm experiment, we show that including information on the dynamics of phenotypic traits such as body size into composite early warning indices can produce more accurate inferences of whether a population is approaching a critical transition than using abundance time-series alone. By including fitness-related trait information alongside traditional abundance-based early warning signals in a single metric of risk, our generalizable approach provides a powerful new way to assess what populations may be on the verge of collapse.

## Abstract (web summary)

Predicting population collapse by monitoring key early warning signals in time-series data may highlight when interventions are needed. Here, the authors show that including information on phenotypic traits like body size can more accurately predict critical transitions than abundance data alone.

## Graphical abstract

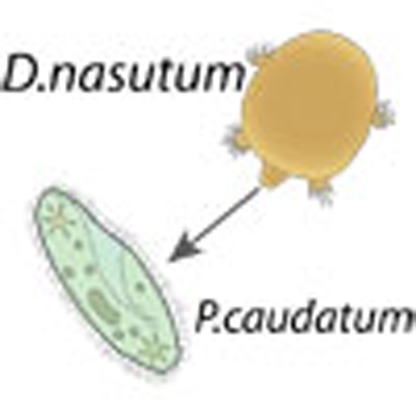

Rapid changes to dynamic systems can lead to the collapse of ecosystems and extinction of species^1^,^2^,^3^,^4^,^5^,^6^. Much recent effort has been invested in predicting such potentially unwanted transitions^7^,^8^,^9^,^10^,^11^,^12^,^13^,^14^, with the eventual aim of providing robust indications of whether a system needs an intervention to prevent it from transitioning to an unwanted state. The forecastability of such events is based on the theoretical prediction that as a system approaches a tipping point statistical signals, so-called ‘leading indicators', embedded within a time series will change predictably^3^,^7^,^11^. This derives from a phenomenon called critical slowing down, whereby the stability of a system, and thus its ability to return to its prior state after a perturbation, decreases due to increasing external pressure forcing the system away from equilibrium^2^. Consequently, changes in leading indicators such as autocorrelation, variance, and the return rate of a time series can be used to determine whether a system is becoming unstable, and thus potentially approaching a tipping point^2^, although see ref. 12.

Most recent work has focused on how the change in one of a number of individual leading indicators can be used as an early warning signal of an approaching tipping point^10^,^15^,^16^,^17^,^18^. This approach searches for a trend in a given leading indicator, typically quantified as the correlation between the leading indicator and time, prior to a regime shift, with strong trends (correlations) being indicative of an approaching transition^10^. This approach has been criticized for being statistically inefficient^9^ and for requiring long and high-quality abundance time-series data^19^, leading to the development of additional methods based on maximum likelihood model selection, more complex state-space models^9^,^14^, as well as early warning signals based on indicators such as the spatial autocorrelation of populations^20^. A alternative and potentially more powerful approach may be the combination of several leading indicators into a single composite metric of risk, which could allow approaching critical transitions to be more reliably inferred^11^. Such an approach has previously been used to combine the trends in coefficient of variation, autocorrelation, skewness, and spatial autocorrelation into a single composite early warning signal, and has been shown to provide better estimates of an approaching transition than single leading indicators alone^11^.

Such abundance-based measures of the stability of a system may not be the only indicators that a population is being stressed; recent studies have shown that demographic change, driven by environmental perturbation, is often accompanied by simultaneous change in individual traits^21^. Such changes in the distribution of fitness-related phenotypic traits are known to be intimately linked to both species' life history and changes in population dynamics^22^,^23^,^24^, and they can be indicative of a population's response to further perturbation. One such trait is body size. Environmental effects on demographic rates are often mediated through body size^25^,^26^, for example, individuals of a population will lose mass as their habitat deteriorates, and subsequently die if habitat quality continues to decline. Further, spatial heterogeneity in resource availability, or individual heterogeneity in access to resources (for example, size-dependent competition), can increase the variation in size within a population in stressful environments^27^,^28^. Thus, shifts in the distribution and value of traits such as body size may in fact precede environmental alterations of the demography of a population. Because body size can substantially influence demography^29^,^30^, these effects at the population level can propagate to larger scales^27^. For example, the body size of species in food webs is known to alter the resilience of the system to disturbance^31^, as well as altering the strength of trophic cascades^32^.

Evidence from the Pleistocene shows that large declines in body size have previously occurred before extinction events^33^, while in harvested systems similar shifts have been shown before large collapses in biomass^34^. Such a pattern is not wholly unexpected, as body size is known to negatively respond to multiple different stressors, including prey availability^35^, climatic change^36^, and pollutants^37^. Given the close relationship between body size, resilience, stressors, and demography, trends in a trait such as body size have the potential to act as additional, and potentially earlier, leading indicator of population collapse. To highlight this, consider a scenario where the size of individuals is directly related to reproduction (larger individuals are able to reproduce more successfully), such that as the size distribution of the population shifts, so too does the growth rate of that population. As the population is stressed, say by reduced resource availability^11^, the body size (and consequently growth rate), of the population will both decline, eventually leading to the collapse of the population. As a population at carrying capacity can be considered to have gone through a critical transition when the realized non-linear growth rate of a population falls below one (and thus the ability of a system to respond to perturbations diminishes)^11^, in the above scenario a negative trend in the mean body size of a population (and consequently in growth rates) could be used as an alternative measure of whether a system is approaching a bifurcation. Given the noted difficulties in accurately estimating the abundance of wild populations^38^,^39^ and the negative impacts this can have on the reliability of traditional demographic-based early warning signals^40^, using alternative measures of stability (such as body size trends) in parallel to traditional abundance-based leading indicators has considerable merit. This will be especially true if estimates of trends in body size are less prone to error than abundance data.

In this study we investigated whether including trait dynamics into composite early warning signals provided more robust and/or earlier estimates of whether populations were at risk of collapse than early warning signals based on abundance time series alone. We analysed data from an experimental ciliate predator–prey system composed of *Didinium nasutum* feeding on *Paramecium caudatum*. Populations of *D. nasutum* were forced through a bifurcation due to varying rates of decline in prey availability. This was likely to be a transcritical bifurcation (a non-catastrophic transition from a small population size to extinction); however, we cannot rule out the possibility that some populations passed through a fold or saddle-node bifurcation (a catastrophic transition where a minimum prey density is required to maintain predator populations, below which, because of Allee effects, predator populations will crash)^41^. The small-scale nature of this system allowed high temporal resolution data on both the abundance and the body size of individuals of *D. nasutum* (the species of interest) to be collected. We then inferred which populations went through critical transitions, and subsequently constructed and tested 127 different composite early warning signals that comprised both trait-based and non-trait-based leading indicators. These 127 composite early warning signals were constructed from every possible combination of one to seven leading indicators of population collapse (that is, 7 composite metrics based on a single leading indicator, 21 based on two leading indicators, and so on). Five of these indicators were those previously identified in abundance time-series data^10^: autocorrelation at the first lag (‘acf,' similarity of value at time *t* to the value at *t*−1), density ratio (‘dr', ratio of low frequencies to high frequencies), first-order autoregressive coefficient (‘ar1', an alternative measure of autocorrelation), return rate (‘rr', rate of return to equilibrium after a perturbation, calculated as 1/ar1) and the coefficient of variation of the data (‘cv'). These five so-called ‘generic' leading indicators have been shown to precede both transcritical and saddle-node bifurcations in a variety of dynamic systems^3^. Two further indicators included in the composite early warning signals were trait based: mean body size (‘size'), and s.d. of body size (‘size.s.d.'). Drake and Griffen^11^ proposed a methodology for combining multiple leading indicators into a single composite early warning metric, where the time series for each leading indicator is normalized at every time point by subtracting the long-run average of that indicator, and dividing it by the long-run s.d. These standardized time series can then be summed together to produced a composite time series that incorporates one or more leading indicators. Such a composite early warning metric is considered to produce an early warning signal value of the composite metric exceeds its running mean by either 1 or 2*σ* (ref. 11). We then assessed the robustness of each composite metric by comparing the proportion of false-positive to true-positive early warning signals generated.

We present evidence that suggests that the inclusion of fitness-related trait dynamic information into early warning metrics can not only provided significantly more reliable early warning signals than more traditional methods but also generate reliable early warning signals earlier than using abundance time series data alone. This novel parallel time-series approach has the potential to increase the poor predictive accuracy of traditional abundance-based early warning signals, potentially allowing us to overcome the temporal and spatial limitations of data available on organisms of conservation concern^19^.

## Results

### Experimental population dynamics

**Figure 1:**
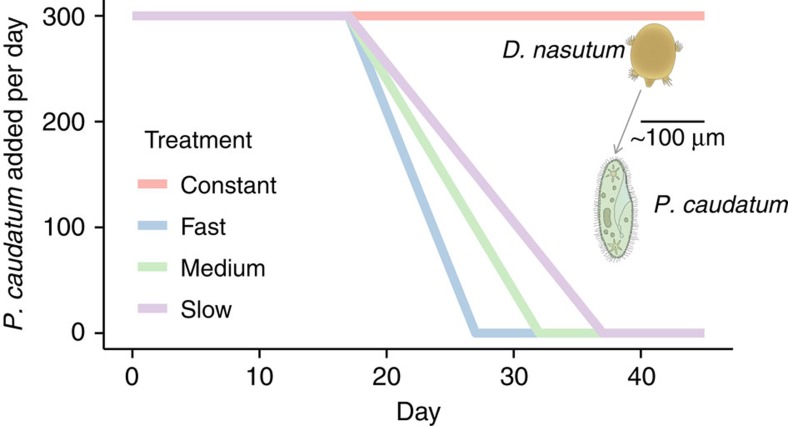
Experimental declines in prey availability. Populations of the predatory ciliate *Didinium nasutum* were fed ∼300 individuals of the prey species *Paramecium caudatum* per day for the first 18 days of the experiment. From day 18 onwards, four experimental treatments were initiated where the number of *P. caudatum* fed to the *D. nasutum* populations was manipulated. Vector images of *Didinium* and *Paramecium* were reproduced with permission from originals by Tracey Saxby, IAN Image Library (ian.umces.edu/imagelibrary).

**Figure 2:**
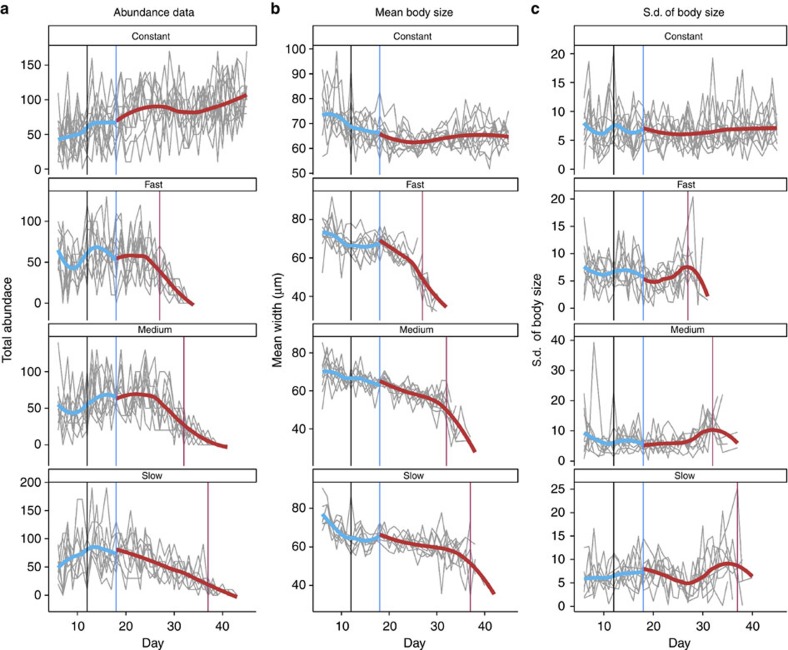
Effects of experimental treatments on *Didinium* abundances and body size. Data on the abundance (**a**) mean body size (**b**) and s.d. of the body size (**c**) of *D. nastrum* populations that were exposed to four different experimental treatments were collected over a period of 45 days. Populations in declining food availability treatments showed significant shifts in body size before population collapse. Black vertical lines indicate the end of the transitory dynamics phase (day 12). Blue vertical lines indicate the start of the experimental treatments (day 18). Red vertical lines indicate the last day that prey were added in each treatment (where applicable). Loess smoothing shows the general responses of populations in each of the four treatments from the start of the monitoring period to the beginning of the experimental treatments (days 6–12, light blue lines), and from the start of the experimental treatments to the extinction of the last experimental population (day 12 onwards, red lines).

The three deteriorating environment treatments (slow, medium, and fast; Fig.1) caused population collapses to occur at varying times, with rapid rates of decline in available prey causing rapid extinction and slow rates of decline leading to prolonged population persistence (Fig. 2a). The experimental treatments also affected how body size changed through time, with rapid changes in prey abundance leading to fast shifts in *Didinium* body size (Fig. 2b), while slow rates of forcing produced large increases in the s.d. of body size (Fig. 2c).

### Effectiveness of composite early warning signals

**Figure 3:**
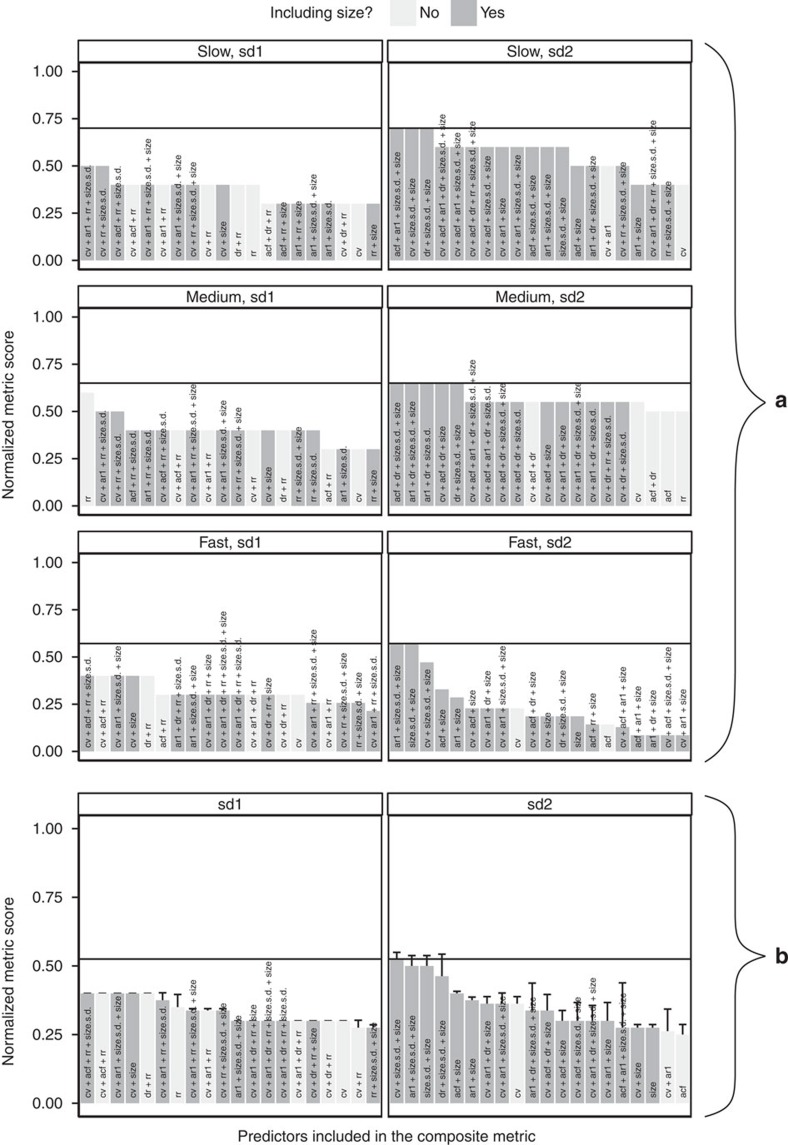
Performance of early warning metrics when including or excluding information on trait dynamics. (**a**) The 20 combined metrics with the highest normalized metric score in each treatment group, and (**b**) across all treatment groups, with an early warning threshold set at one or two s.d.'s. Including size within the early warning index increased the predictive accuracy across all treatments at a 2*σ* threshold. At a 1*σ* threshold including size improved on or matched the accuracy of other combined metrics in all treatments apart from the medium rate of change, where return rate was the most reliable indicator. Horizontal black lines show the highest normalized metric score in each treatment. Bars indicate the variance in predictive accuracy of each metric across the three different deterioating experimental treatments. acf, autocorrelation; ar1, autoregressive coefficient; cv, coefficient of variation; dr, density ration; rr, return rate; size, mean body size; size.s.d., s.d. of body size.

Including body size within composite early warning metrics generally improved the accuracy of predictions, especially at a 2*σ* threshold for early warning signals (Fig. 3a,b). Across the three experimental treatments, population crashes were more accurately forecasted in treatments with slower rates of forcing than with faster rates, with the best metrics having a normalized metric score (proportion of true-positive minus the proportion of false-positives early warning signals) of ∼0.7 in the slow treatment and ∼0.6 in the fast treatment (Fig. 3a). Using a 2*σ* threshold ensured there were less false-positive signals while retaining a high rate of detection of true-positive signals across all three treatments (black horizontal lines, Fig. 3a).

When results from all three experimental treatments were combined, ∼60% of the most reliable metrics at a 1*σ* threshold included trait information, while at a 2*σ* threshold 85% did (Fig. 3b). Of all the metrics tested, a combination of the coefficient of variation, the mean size of the population and the s.d. of the size (cv+size.s.d.+size) was the most robust, and showed relatively low variance between the three experimental treatments (Fig. 3b).

### Timing of early warning signals

**Figure 4:**
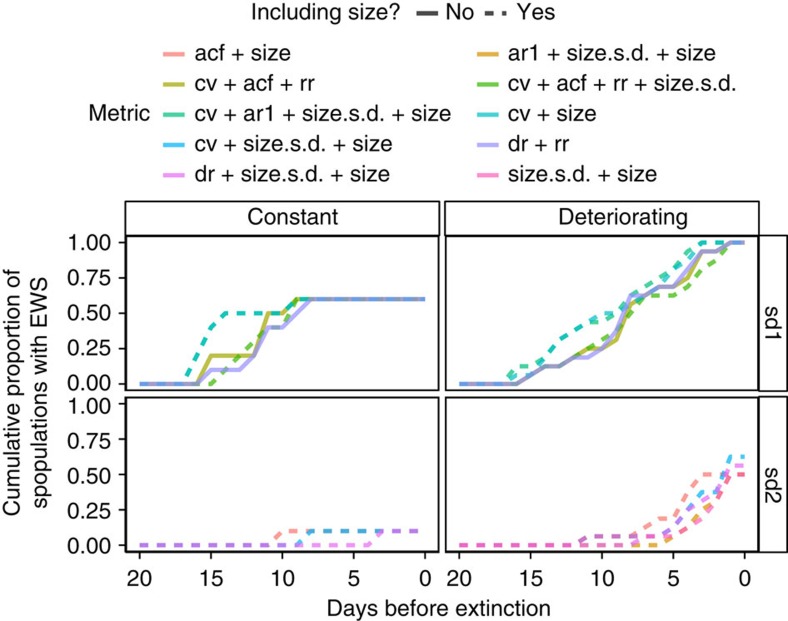
Timing of early warning signals when including or excluding information on trait dynamics. The cumulative proportion of populations in the constant or deteriorating treatments that showed early warning signals with a threshold of either 1 or 2*σ*. The metrics displayed are the five metrics with the highest normalized metric score at 1 or 2*σ* in the constant treatment and across all three of the deteriorating treatments (slow, medium and fast). acf, autocorrelation; ar1, autoregressive coefficient; cv, coefficient of variation; dr, density ration; rr, return rate; size, mean body size; size.s.d., s.d. of body size.

Early warning signals were detectable up to 16 days before a bifurcation using a 1*σ* threshold (Fig. 4b); however, this also resulted in a high proportion of false positives (>50%, Fig. 4a). False-positive rates were similar regardless of whether trait-based indicators were included in the composite metric, but including trait information tended to produce warning signals (both false positive and true positive) earlier than metrics that did not include trait dynamics (Fig. 4a).

With a 2*σ* threshold, the rates of false positives were low (∼10%, Fig. 4c), while the proportion of true positive remained relatively high—up to 65% across the three treatments (Fig. 4d). Including trait information reduced the proportion of false positives (Fig. 4c), and increased the proportion of true positives (Fig. 4d).

## Discussion

Recent work on the detectability of critical transitions has tended to focus on the change in single leading indicators, and how reliable these methods may be in predicting future population crashes^10^,^19^. However, while it has been known for some time that composite early warning metrics can be more reliable for detecting critical transitions than single leading indicators^11^, further development in this area has been limited. The inclusion of data on trait dynamics into composite early warning metrics could provide a paradigm shift in the approaches taken to predict critical transitions, potentially allowing shorter, more realistic, time series to be reliably analysed^19^.

Our results strongly suggest that including data on fitness-related phenotypic traits, such as body size, could significantly improve our ability to predict regime shifts before their occurrence. Not only does including trait information produce fewer false-positive signals, but, depending on the threshold value, it can also make true-positive signals detectable earlier, 11 days before a bifurcation with a 2*σ* threshold. The benefit gained by including traits within early warning metrics is necessarily relative to the strength of the change of the trait in response to the pressure exerted on the system. Our experimental populations showed significant changes in mean body size and the s.d. of body size as the habitat quality degraded (Fig. 2), which explains the relatively good performance of composite metrics including one or more measures of the change in body size of a population (Figs 3 and 4). Less extreme changes in trait values will decrease the importance of including trait data within a composite metric. Clearly before a trait, be that body size or some other characteristic, should be considered an appropriate candidate to be included within a composite metric a theoretical expectation that the trait should respond to the forcing of the system is required. Whether a trait responds to such forcing will depend not only upon the identity of the trait, but also on the nature of the forcing itself.

That being said, body size or mass are obvious candidate traits to include within early warning signals, as body size has been shown to have significant impacts on interaction strengths^42^, the nature of trophic cascades^32^, the structure of food webs^31^ and the extinction risk of species^43^. In addition, body mass can be plastic in response to environmental conditions^44^,^45^, and thus could act as an indicator of environmental degradation and stress on a system. Previously, such measurements of this plastic phenotypic trait have been shown to provide a cumulative summary of a population's past environmental experience and, thus act as a ‘memory statistic'. Here we shown that this can be used to better predict population's response to future environmental conditions. Finally, because of the broad suit of research into the effects of these phenotypic traits, data on body size and mass are more readily available than many other traits.

Our results suggest that, for time series of similar lengths to those tested here, a combined metric based on the coefficient of variation of the abundance time series, and trends in the mean body size and s.d. of the body size with a 2*σ* threshold may produce the most robust signals of population collapse in real world systems. However, because the data analysed here are not necessarily representative of all population crashes or regime shifts this result must be considered preliminary. We suggest that identifying which potential composite metrics are most robust to, say, the noisy nature of survey data, or the complexity of the system, should be a critical next step in making such methods widely applicable^19^.

Although populations responded somewhat differently to the three deteriorating environment treatments (Fig. 2), there were not large differences in the performance of the combined metrics across the treatment groups (Fig. 3b). Generally, metrics performed better in the slow and medium treatments than the fast; however, the conclusions from the three treatments were the same: including trait dynamic information consistently improves overall predictive accuracy, and a 2*σ* threshold provides a better ratio of false-positive to true-positive early warning signals than a 1*σ* threshold (Fig. 3b). However, while previous work has used a simple threshold value of 1 or 2*σ* to indicate whether an early warning signal is present^11^, this approach becomes untenable when the time series being tested are long, as even in a stable stochastic system a leading indicator will pass its long-run mean by a 2*σ* threshold value given enough time. One potential solution to this issue would be to set a threshold value that is dependent on the length of the time series being tested, with longer time series requiring a higher threshold value to generate an early warning signal; however, assessing the utility of such an approach is beyond the scope of this manuscript.

In conclusion, we demonstrate that the inclusion of trait dynamic information into composite metrics for detecting early warning signals has the potential to significantly increase the predictive power of such approaches. The method we use is flexible enough that any trait or combination of traits can be included simultaneously, alongside more traditional early warning signals. Using such an approach, we were able to reliably detect approaching tipping points up to 10 days (≈20 generations^46^) before their occurrence. This composite approach performs significantly better than single leading indicators alone, and could provide a methodology for more accurately forecasting the collapse of wild populations.

## Methods

### Experimental data

Data on population collapses were generated using an experimental microcosm set-up with 60 replicate populations (15 per treatment, see below) of the predator–prey system *Didinium nasutum* (predator) feeding on *Paramecium caudatum* (prey). Manipulating the prey availability through time drove collapse of the predator populations.

Microcosms consisted of 15-ml plastic tubes, with 10 ml of sterile Chalkley's medium^47^ into which ∼300 individuals of the bactiverous ciliate prey species *P. caudatum* were added. Preliminary experiments suggested that populations of *D. nasutum* could readily consume 300 *P. caudatum* over a 24-h period, meaning that prey densities would not increase through time. *D. nasutum* populations were established by adding approximately three individuals to each of the microcosms (experimental day 0). Approximately, 300 individuals of *P. caudatum* were added to each microcosm every 24 h until the declining prey availability treatments were started.

No sampling occurred from days 0–6, to allow *D. nasutum* to reach high densities (approximately five individuals per millilitre). After this period, 1 ml of the media of each microcosm was removed every 24 h until the end of the experiment, and the number of *D. nasutum* counted by eye using a stereoscopic microscope. The sample was put through a continuous imaging flow cytometer (FlowCAM), and the widths of the *D. nasutum* individuals quantified digitally. Width was used as a measure of body size because, given the approximately cylindrical shape of *D. nasutum*, no matter what the orientation of the individual protozoan when the measurement was made the width of the individual was always constant. The sample was then discarded to allow prey to be added without increasing the volume in which the *D. nasutum* population resided. Any deficit in volume after the prey had been added was topped up with sterile Chalkleys medium to ensure microcosms had a fixed volume.

Populations had appeared to stabilize by day 12, and thus the transitory dynamics of the system were considered to be over. Declining food treatments were initiated on day 18. The microcosms were split into four treatment groups as follows: (1) constant; (2) slow; (3) medium; and (4) fast. In the constant treatment, populations continued to be fed 300 prey individuals per day for the full length of the experiment (45 days). The three treatments where food availability declined (slow, medium and fast) did so at different rates. In the slow treatment, the amount of prey declined linearly from 300 *P. caudatum* per day to 0 per day over a 20-day period, for the medium this occurred over a 15-day period and for the fast over a 10-day period. These differing rates of decline of the available prey led to distinctly different pre-extinction population dynamics, and changes in the body size of *D. nasutum* (Figs 1 and 2). Microcosms that were subjected to declining food resources were sampled every day until no individuals were counted in the 1 ml sample for 2 consecutive sampling days. Extinction was assumed to have occurred on the first of these days. The experiment ended when all populations in the declining food treatments were extinct (day 45). Of the 15 replicate *D. nasutum* initially set-up in each treatment, several failed to establish during the first 6 days and were abandoned, leaving 11 replicates in the constant treatment, 9 in the fast, 9 in the medium and 8 in the slow. Experimental data are included with this publication (Supplementary Data 1).

### Composite early warning signals

**Figure 5:**
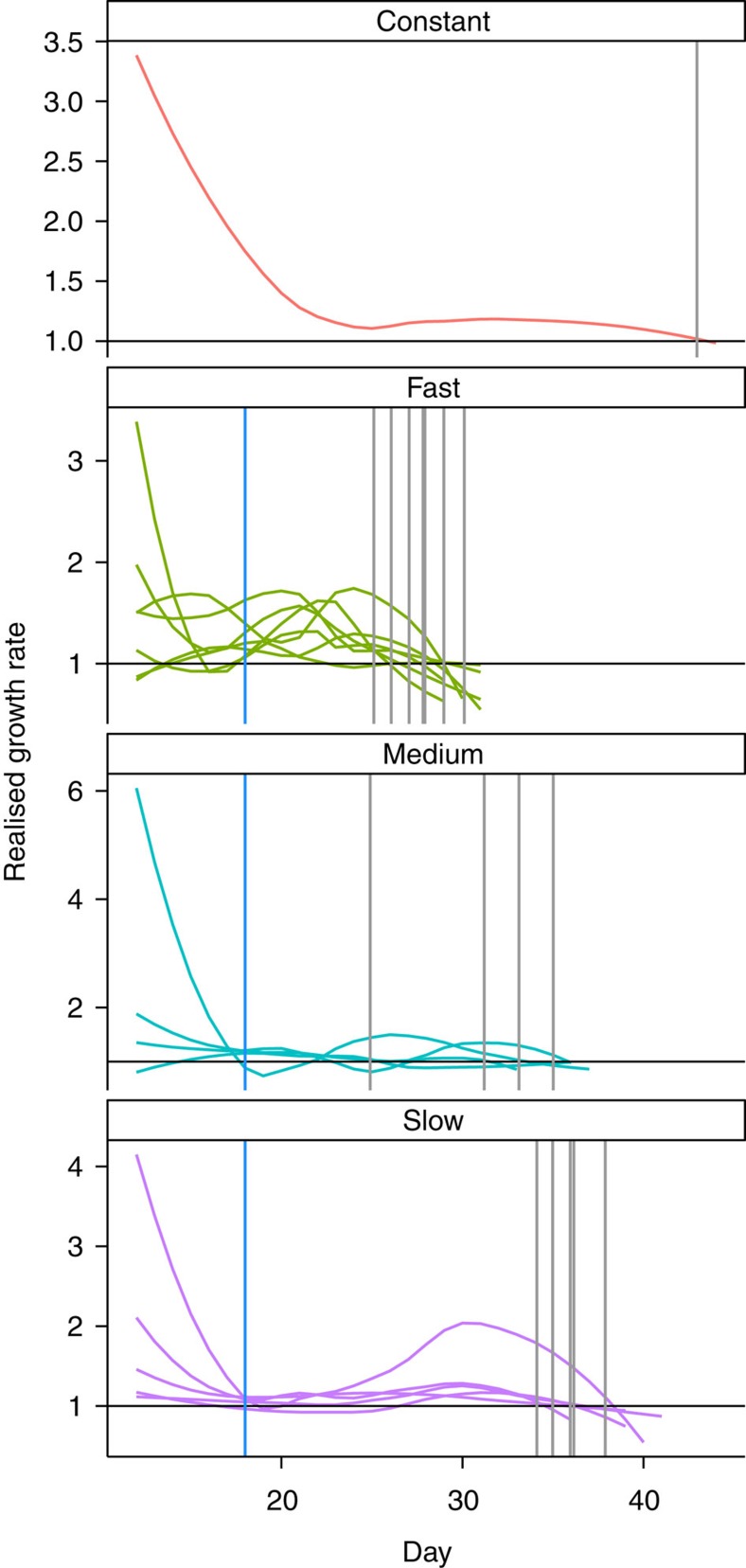
Realized population growth rates in successive time intervals for each population across the four experimental treatments. For general density-dependent growth in a deteriorating environment, a bifurcation occurs when the non-linear growth rate drops below 1. Thus, we inferred a bifurcation to have occurred at the last time before extinction of a population that the realized growth rate dropped below 1 (grey vertical lines). One population in the constant environment treatment, seven in the fast degrading environment, four in the medium and five in the slow treatments appeared to cross this threshold. Vertical blue lines indicate the start of the declining food treatments (day 18, where applicable).

Each experimental population was analysed separately, and only data from day 12 onwards were used in the analysis to exclude data on transitory dynamics^11^ (Fig. 2). For general density-dependent growth, a bifurcation occurs when the non-linear growth rate falls, and remains, below 1 (ref. 11). Therefore, for each population, we calculated the realized growth rate in successive time intervals, and used loess smoothing to establish when realized growth rates drop below 1 as in ref. 11 (Fig. 5; Supplementary Data 2).The time of a bifurcation was recorded as the first point at which the realized growth rate dropped and stayed below 1 before the extinction of a population. Not all experimental replicates in the deteriorating treatments were inferred to have passed through a bifurcation, likely due to additional stochasticity in population estimates generated by the necessity of counting individuals in a subsample (10%) of the microcosms. We only analysed populations that were inferred to have gone through a bifurcation, and excluded from the subsequent analysis one population of the constant treatment that had appeared to cross this bifurcation (Fig. 5).

For the fast, medium and slow treatments, we analysed the final 20 time points before each bifurcation, thus normalizing the length of each time series and minimizing any effect of varying time-series length. For the constant treatment populations, we analysed the 20 time points from day 12 to 32, which allowed us to guarantee that no population collapse occurred after the data analysed, and thus any early warning signals generated in the constant treatment populations were false positives.

Composite early warning signals were calculated based on the method proposed by Drake and Griffen^11^. In all, we tested 127 different composite early warning signals, composed of every unique combination of one to seven leading indicators. Of the seven different leading indicators used, five are considered to be generic to many dynamic systems and are a product of critical slowing down^10^,^11^: (1) autocorrelation at the first lag, (2) density ratio, (3) first-order autoregressive coefficient, (4) return rate and (5) the coefficient of variation of the data. While several of these indicators are mathematically very similar (notably, autocorrelation at first lag, the first-order autoregressive coefficient, and return rate), when data are noisy these leading indicators have been shown to perform differently^19^. The two other indicators included within the composite early warning signals were trait based: body size (‘size') and the s.d. of body size (‘size.s.d.'). It should be noted that one would not expect classic early warning signals (for example, increasing autocorrelation and decreasing return rate) to be present in the body size data (as would be expected in the population abundance data). Rather, we argue that one would expect a consistently declining trend before collapse in the mean body size, and, potentially, an increasing trend in the variance of body size.

**Figure 6:**
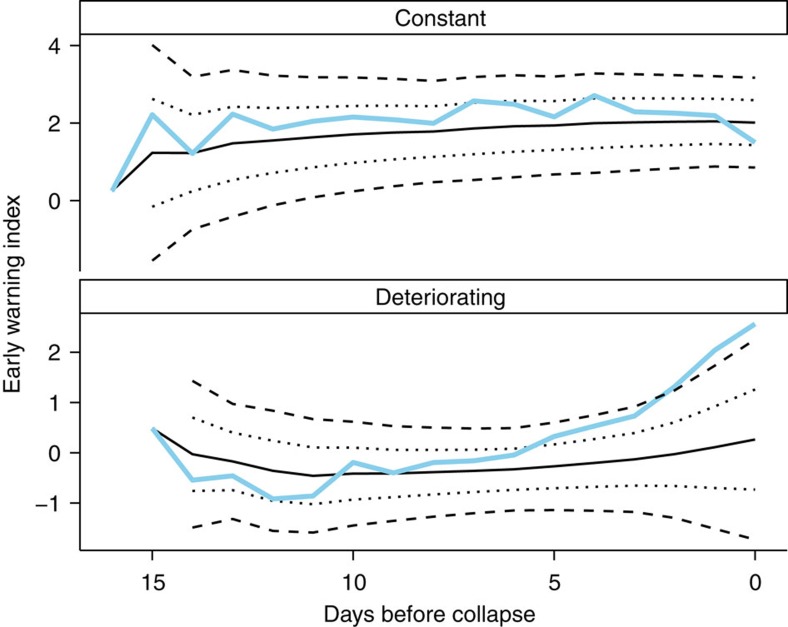
How the threshold value determines whether an early warning signal is present. As in Drake and Griffen, an early warning signal was considered to be generated when the value of the early warning index (blue lines) exceeded its running mean (solid black lines) by either 1 (dotted black lines) or 2*σ* (dashed black lines). This is highlighted above with an example calculated for a single a population from each of the constant and deteriorating experimental treatments.

Each of these leading indicators was calculated for each experimental population individually, and at each day observations were made during the experiment (Supplementary Data 3). As in Drake and Griffen^11^, we normalized each leading indicator by subtracting the long-run average of that indicator from the value of that indicator at time *t*, and divided it by the long-run s.d.. Thus, each statistic at time *t* 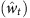 was calculated as


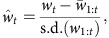


where 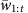 is the mean of a statistic from times 1 to *t*, and s.d. (*w*_1:*t*_) is the s.d. over the same period. To calculate the composite early warning signal, the values for the leading indicators to be included were summed at each time point, giving a time series of the composite early warning signal (Fig. 6). Body size and return rate were both expected to decline prior as a population approached a tipping point, body size because of the decreasing food resources and return rate because as a system becomes more unstable it takes longer to return to its equilibrium after a perturbation. Consequently, the normalized values of body size and return rate (as calculated above) were multiplied by −1 so they could be included within the composite early warning signal. An early warning signal was consider as present if at any point the value of this composite leading indicator exceeded its running mean by either one or two s.d.'s (Fig. 6).

We present the results of these analyses in two ways. The first is the normalized metric score. This was calculated by subtracting the proportion of false-positive signals produced by each composite metric (early warning signals produced using data from the constant treatment) from the proportion of true-positive signals produced by each composite metric (using data from each of the deteriorating environment treatment groups; Fig. 3). This gives an overall idea of how well each of the metrics performs across the different treatment groups, and whether a 1 or 2*σ* threshold produces more accurate results.

The second approach is to present the cumulative proportion of populations that showed early warning signals through time (Fig. 4). The first occurrence of an early warning signal in each population (where present) was recorded, and the cumulative proportion of these through time was calculated, with data grouped into constant and deteriorating (slow, medium and fast together) treatments to simplify the interpretation of the results. This indicated which of the composite metrics warned earliest of an approaching collapse, and whether including trait-based signals in the composite indicators made early warning signals detectable earlier.

All analyses were carried out using the statistical software R^48^ (Supplementary Data 2 and 3).

## Additional information

**How to cite this article:** Clements, C. F. & Ozgul, A. Including trait-based early warning signals helps predict population collapse. *Nat. Commun.* 7:10984 doi: 10.1038/ncomms10984 (2016).

## Supplementary Material

Supplementary Data 1 Data from the protist microcosm experiment analysed for early warning signals.

Supplementary Data 2 R code to calculate the realised growth rates of each population, and thus the time at which each population goes through a critical transition.

Supplementary Data 3 R code to calculate the composite early warning signals and plot the results of the analysis.
